# Single-cell RNA-seq highlights heterogeneity in human primary Wharton’s jelly mesenchymal stem/stromal cells cultured in vitro

**DOI:** 10.1186/s13287-020-01660-4

**Published:** 2020-04-06

**Authors:** Changbin Sun, Lei Wang, Hailun Wang, Tingrun Huang, Wenwen Yao, Jing Li, Xi Zhang

**Affiliations:** 1 BGI Education Center, University of Chinese Academy of Sciences, Shenzhen, 518083 China; 2 BGI-Shenzhen, Shenzhen, 518083 China; 3 China National GeneBank, BGI-Shenzhen, Shenzhen, 518120 China; 4 James D. Watson Institute of Genome Science, Hangzhou, 310008 China; 5 Department of Radiation Oncology, School of Medicine, Johns Hopkins University, Baltimore, MD 21218 USA

**Keywords:** MSCs, scRNA-seq, Highly variable genes, Functional enrichment, Potency

## Abstract

**Background:**

Mesenchymal stem/stromal cells (MSCs) are multipotent cells with a promising application potential in regenerative medicine and immunomodulation. However, MSCs cultured in vitro exhibit functional heterogeneity. The underlying molecular mechanisms that define MSC heterogeneity remain unclear.

**Methods:**

We investigated the gene expression profile via single-cell RNA sequencing (scRNA-seq) of human primary Wharton’s jelly-derived MSCs (WJMSCs) cultured in vitro from three donors. We also isolated CD142^+^ and CD142^−^ WJMSCs based on scRNA-seq data and compared their proliferation capacity and “wound healing” potential in vitro. Meanwhile, we analyzed publicly available adipose-derived MSC (ADMSCs) scRNA-seq data and performed transcriptome comparison between WJMSCs and ADMSCs at the single-cell level.

**Results:**

GO enrichment analysis of highly variable genes (HVGs) obtained from WJMSCs revealed that these genes are significantly enriched in extracellular region with binding function, involved in developmental process, signal transduction, cell proliferation, etc. Pathway analysis showed that these HVGs are associated with functional characteristics of classic MSCs, such as inflammation mediated by chemokine and cytokine signaling, integrin signaling, and angiogenesis. After regressing out the batch and cell cycle effects, these HVGs were used for dimension reduction and clustering analysis to identify candidate subpopulations. Differentially expressed gene analysis revealed the existence of several distinct subpopulations of MSCs that exhibit diverse functional characteristics related to proliferation, development, and inflammation response. In line with our data, sorted CD142^+^ and CD142^−^ WJMSCs showed distinct proliferation capacity as well as “wound healing” potential. Although WJMSCs and ADMSCs were derived from different tissues and were displaying different differentiation potencies, their HVGs were largely overlapped and had similar functional enrichment.

**Conclusion:**

HVGs identified in MSCs are associated with classic MSC function. Regarding therapeutic potential, these genes are associated with functional characteristics, on which the MSC clinical application were theoretically based, such as development and inflammation response. Altogether, these HVGs hold the potential to be used as candidate markers for further potency association studies.

## Background

Mesenchymal stem/stromal cells (MSCs) are multipotent with self-renewal capacity and can be derived from various tissues, including the bone marrow [1], adipose tissue [2], umbilical cord [3, 4], and placenta [5]. Besides the multi-lineage potential to differentiate into various cell types, such as chondrocytes, osteocytes, adipocytes, myocytes, and neuronal cells [6, 7], MSCs could modulate immune cell response via interaction with lymphocytes from both the innate and adaptive immune system to deliver immunosuppressive and anti-inflammatory effects after homing to sites of inflammation in vivo [8, 9]. Furthermore, human MSCs could be cultured in large scale and have minimal functional loss after long-term cryopreservation [10, 11]. Therefore, MSCs demonstrate promising utilization potential and are ideal cell types in both fundamental and translational biology fields, such as developmental biology, cellular therapy, immunomodulation, and regenerative medicine [12, 13]. Currently, more than 700 clinical trials have been registered in ClinicalTrials.gov (http://www.clinicaltrials.gov), which utilize MSCs for cellular therapy. Transplantation of MSCs demonstrates no obvious adverse effect, regardless of allogeneic or autologous cell origin, and has been extensively explored in treatment of various disease types, such as bone and cartilage defects [14, 15], cardiovascular disease [16, 17], neurological degeneration [18, 19], liver disorder [20], and immunological diseases [21, 22] with encouraging clinical outcomes. Several MSC-based products have been approved or conditionally approved in certain country or district to treat disorders, such as graft versus host disease (GvHD) and Crohn’s related enterocutaneous fistular disease [23].

The minimal criteria for defining multipotent MSCs were published by the International Society for Cellular Therapy (ISCT) in 2006 [24], which is widely accepted and adopted in both basic research and industrial application. However, it only defines basic morphological and functional characteristic. More and more research works have recognized that MSC populations exhibit tissue-to-tissue functional variation [25, 26], as well as inter-population heterogeneity when using current markers to define MSCs, which makes it difficult to predict cell population dynamics and functional alterations after extended culture or exposed to extrinsic factors [27, 28]. Functional heterogeneity coupled with large-scale expansion in clinical manufacturing process may explain, in part, why data across MSC-based clinical trials are largely incongruent [29]. During MSC culturing and passaging, the competition and balance between different subpopulations may change, resulting in decrease in proportion or even loss of certain subpopulations and ultimately leading to alterations in cell function and treatment outcomes in clinical studies [30, 31]. Therefore, there is an urgent need to elucidate whether a certain MSC subtype or a cocktail of defined population of different subtypes can demonstrate effectiveness during cellular therapy and tissue engineering. Investigation into the underlying molecular mechanisms that define MSC heterogeneity will facilitate subtype identification and improve methods for cell isolation and expansion in vitro. By in-depth analysis of cell quality attributes, it will also help to interpret the results from clinical trials and eventually improve clinical efficacy of MSC products.

Recently, single-cell RNA sequencing (scRNA-seq) technology, which allows massive parallel analysis of gene expression profiles at single-cell level, has become a powerful tool in investigating tissue and cell heterogeneity. It provides unprecedented opportunities for identifying subpopulations that share a common gene expression profile in a heterogeneous cell population [32]. Here, we investigated the gene expression profile via scRNA-seq of human primary WJMSCs cultured in vitro from three donors. In contrast to other source-derived MSCs, WJMSCs, which isolated from previously discarded umbilical cord tissues, bear higher proliferation rate and the strongest immunomodulatory effect, making them an attractive alternative source of MSCs for clinical research and application [33, 34]. Meanwhile, we analyzed publicly available ADMSC scRNA-seq data [35] and performed transcriptome comparison between WJMSCs and ADMSCs at the single-cell level.

## Methods

### Cell isolation and culture

Human umbilical cord tissues were collected from naturally delivered full-term newborns (*n* = 3, two females and one male). WJs were isolated from the umbilical cord after dissection and mechanically dissociated into tissue explants of approximately 2 mm^2^, which were then seeded into T75 flasks and cultured in UltraCULTURE™ Serum-free Medium (LONZA) at 37 °C with 5% CO_2_ in a humidified atmosphere. After the cell density reached about 80% confluence, cells were dissociated with TrypLE™ Select (Thermo Fisher Scientific) incubated at 37 °C for 5 min. The collected cells were immediately used for single-cell library construction, sub-cultured into a new culture dish, tri-lineage differentiation potency test, or freezing in liquid nitrogen for long-term banking.

### scRNA-seq and analysis

scRNA-seq experiment was performed using the Chromium Single Cell Gene Expression Solution, V2 Chemistry (10x Genomics), following the manufacturer’s protocol. Briefly, the collected cells were washed with PBS twice and resuspended in 500 μl PBS, targeting the required 500 cells/μl concentration. We pipetted 6.4 μl cell suspension, targeting the recovery of about 2000 cells per sample. Single-cell RNA-seq libraries were obtained following the 10x Genomics recommended protocol, using the reagents included in the Chromium Single Cell 3′ v2 Reagent Kit. Libraries were sequenced on the BGISEQ-500 (BGI) instrument [36] using 26 cycles (cell barcode and UMI [37]) for read1 and 108 cycles (sample index and transcript 3′ end) for read2.

scRNA-seq data analysis was available in (Additional file 1) for details. Briefly, the scRNA-seq data was processed using cellranger-2.0.0 for mapping, outlier cells using the median absolute deviation from the median total library size (logarithmic scale), and total gene numbers (logarithmic scale), as well as mitochondrial percentage, as implemented in scran package, using a cutoff of 3 [38]. Any gene expressed across all the cells by average UMI less than 0.1 was removed. Cell cycle phase assignment and confound effect removal, highly variable gene identification, linear and nonlinear dimension reduction, and clustering and differential expression analysis were performed using the Seurat package [39].

### Bulk RNA-seq and analysis

Total mRNA was extracted using the TRIzol (Invitrogen) reagent, as described previously [40]. All protocols for BGISEQ-500 library construction, preparation, sequencing, and quality control were provided by BGI.

Reads were mapped to the human genome (GRCh38) using HISAT2 with default parameters [41]. Raw counts of sequencing reads for the feature of genes were extracted by featureCounts [42]. Transcripts per million (TPM) [43] was used to normalize for sequencing depth and gene length for relative quantity, and then we ranked the gene abundance according to the TPM values. SRA files of bulk RNA-seq data of MSCs derived from adipose tissues (GEO: GSE76081) were downloaded from NCBI, and the same processing pipeline was used for read mapping and normalization.

### Functional enrichment analysis

GO-slim and protein class and pathway overrepresentation test enrichment analyses were performed using PANTHER™ Version 14.1 according to Mi et al. [44] via a test type of Fisher’s exact, applying the Benjamini–Hochberg false discovery rate (FDR) correction for multiple testing.

### Lineage differentiation potency evaluation

Using the marker genes listed in (Additional file 2: Table S1), we calculated the osteogenic, adipogenic, chondrogenic, neurogenic, and myogenic “scores” according to [45]. Specifically, the score was defined as a single numeric value representative of the expression of multiple marker genes and the sum of log normalized expression across all markers in a category. Housekeeping genes were also used and named as “housekeeping score.”

### Tri-lineage differentiation

For osteogenic differentiation, MSCs were seeded into a 24-well plate at the density of 5 × 10^3^ cells/well. When the cells reached 70% confluency, the medium was replaced with osteogenic differentiation medium (MEM-alpha medium (Thermo Fisher Scientific), 10% FBS (Hyclone), 1% Pen-Strep (Thermo Fisher Scientific), 100 nM dexamethasone (Sigma), 10 mM sodium β-glycero phosphate (Sigma), 0.05 mM ascorbic acid (Sigma)) and kept for 3 weeks. To assess the osteogenic differentiation, Alizarin Red S staining (Sigma) was performed for the calcium-rich extracellular matrix.

For adipogenic differentiation, 1 × 10^4^ cells were seeded per well. The cells at confluence were then treated with adipogenic differentiation medium (high-glucose DMEM (Thermo Fisher Scientific), 10% FBS (Hyclone), 0.5 mM IBMX (Sigma), 1 μM dexamethasone (Sigma), 1.7 μM insulin (Sigma), 0.2 mM indomethacin (Sigma)) for 3 weeks. The cells were fixed with 4% formaldehyde solution, and lipid droplets of the resultant differentiated cells were detected using Oil Red O staining (Sigma).

For chondrogenic differentiation, the StemPro™ Chondrogenesis Differentiation Kit was used and performed according to the manufacturer protocol. Briefly, 1.6 × 10^7^ cells were resuspended in MSC medium. Micromass cultures were generated by seeding 5-μL droplets of cell solution in the center of 96-well plate wells. After cultivating micromass cultures for 2 h under high humidity conditions, we added warmed chondrogenesis media to culture vessels and incubated them in 37 °C incubator with 5% CO_2_. After 3 weeks, the cells were fixed with 4% formaldehyde solution and stained with Alcian Blue (Sigma).

### FACS-based cell sorting

For CD142^−^ and CD142^+^ cells isolation, MSCs were stained for 30 min on ice with PE mouse anti-human CD142 antibody (BD Biosciences). PE mouse IgG1 and κ Isotype Control (BD Biosciences) were used as an isotype control. Then, the stained cells were washed, analyzed, and sorted on a BD FACSAria II (BD Biosciences).

### Real-time quantitative polymerase chain reaction (real-time qPCR)

Total RNA was isolated using TRIzol™ Reagent (Invitrogen). PrimeScript™ RT reagent Kit with gDNA Eraser (Perfect Real Time) (TAKARA) and TB Green® Premix Ex Taq™ II (Tli RNAseH Plus) (TAKARA) were used for cDNA synthesis and qPCR according to manufacturer’s protocol, respectively. Applied Biosystems StepOnePlus Real-Time PCR System (Thermo Fisher Scientific) was used for data collection and analysis. Data were representative of three independent experiments with two replicates for each and normalized to inner reference GAPDH. The primers for real-time qPCR are listed in Table 1.

**Table 1:** List of primers for real-time qPCR

Name	Forward primer (5′-3′)	Reverse primer (5′-3′)	Size (bp)
TGFB1	CAATTCCTGGCGATACCTCAG	GCACAACTCCGGTGACATCAA	86
SPARC	AGCACCCCATTGACGGGTA	GGTCACAGGTCTCGAAAAAGC	105
COL4A1	GGGATGCTGTTGAAAGGTGAA	GGTGGTCCGGTAAATCCTGG	113
COL1A1	GTGCGATGACGTGATCTGTGA	CGGTGGTTTCTTGGTCGGT	119
COL5A1	TACCCTGCGTCTGCATTTCC	GCTCGTTGTAGATGGAGACCA	97
CCL2	GATCTCAGTGCAGAGGCTCG	TGCTTGTCCAGGTGGTCCAT	153
CXCL8	ACTGAGAGTGATTGAGAGTGGAC	AACCCTCTGCACCCAGTTTTC	112
MKI67	GCCTGCTCGACCCTACAGA	GCTTGTCAACTGCGGTTGC	127
GAPDH	CTGGGCTACACTGAGCACC	AAGTGGTCGTTGAGGGCAATG	101

### Wound healing assay

Wound healing assays were performed as previously described [46]. Briefly, when sorted MSC cultures were approximately 70% confluent, serum-free DMEM/F12 medium supplemented with insulin, transferring, selenium (ITS-X, Invitrogen), and antibiotics were used to substitute the MSC medium. After 72 h, the conditioned medium was collected and filtered (0.2 μ filter) to remove all cellular debris. Human skin fibroblasts were seeded into 24-well plates and cultured at 37 °C and 5% CO_2_ to form a confluent monolayer. Sterile pipette tips were used to scratch a “wound field.” Then, the conditioned medium was added to the culture. Wound closure was monitored by collecting digitized images (Olympus CKX41) after the scratch was performed. ImageJ was used to obtain the wound area for each image. Data were presented as percentage of wound area compared to the original scratch for each given time point.

## Results

### Overview of the single-cell RNA sequencing data

To investigate into inter-population heterogeneity in primary cultured WJMSCs at the single-cell transcriptome level, primary cells isolated from three human umbilical cords (named as UC1, UC2, and UC3, respectively) were collected and used for scRNA-seq. A total of about 5 × 10^8^ raw reads with high quality for each donor was obtained (Additional file 3: Figure S1A). Mapping these reads to human GRCh38 genome, an average of about 56.90% and 61.03% reads was mapped confidently to the transcriptome and exonic regions, respectively (Additional file 3: Figure S1B, C). Briefly, a total of 6878 cells (filtered matrix) was obtained from the three donors, with an average of 2293 cells for each, with 209,769 mean reads, 38,983 median unique molecular identifier (UMI) counts, and 6279 median genes per cell (Additional file 3: Figure S1D-F), suggesting that our data were of high quality.

**Fig. 1:**
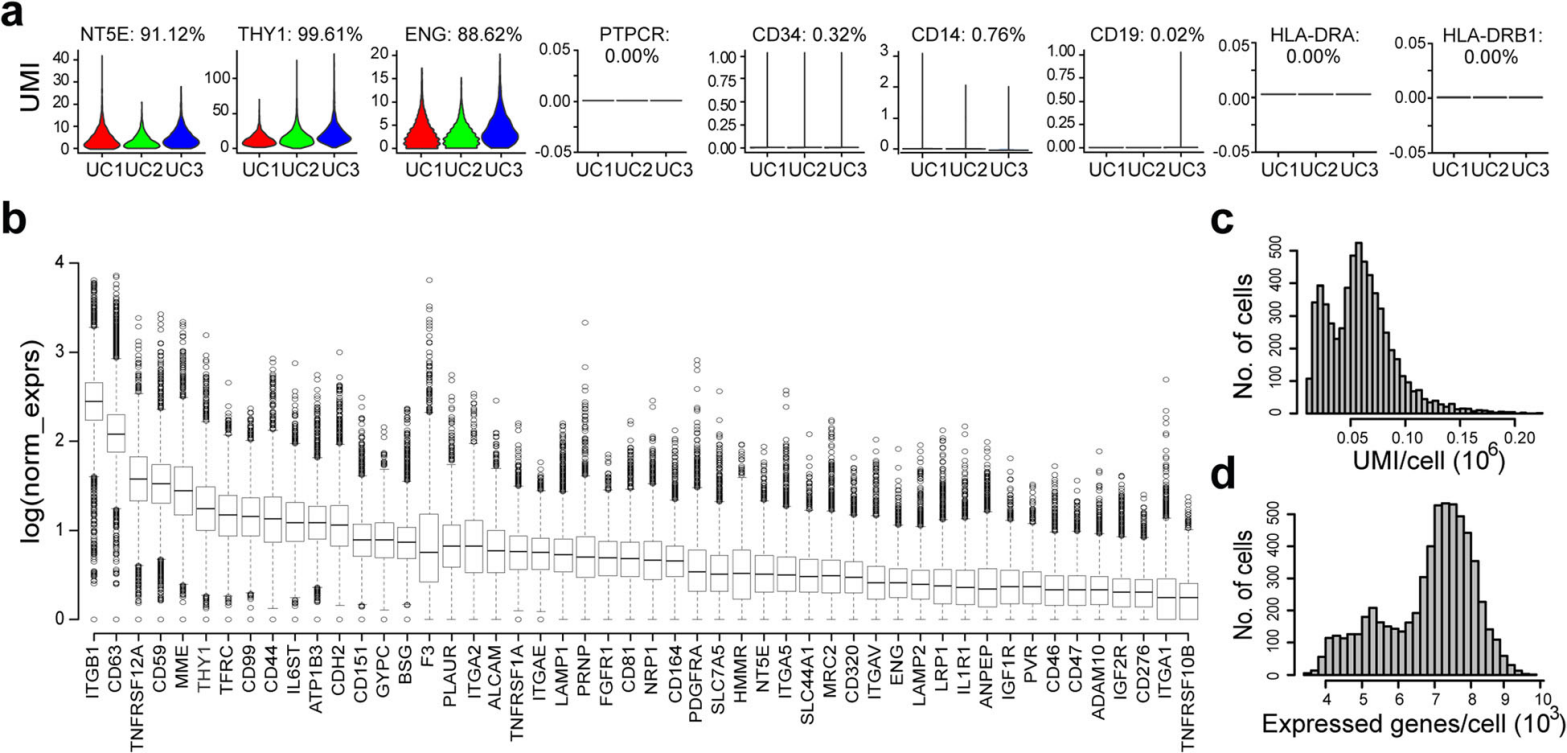
Overview of WJMSCs single-cell RNA-seq data. **a** Expression of marker genes in the three samples. Number on the top showing percentage of cells with at least one UMI. **b** Boxplot showing top 50 cluster of differentiation (CD) genes ranked by average normalized expression. **c** Distribution of UMI cross cells after pre-processing to filter out low-quality cells. **d** Distribution of expressed genes after pre-processing to filter out low-abundance genes with mean-based method (genes with means more than 0.1 were retained)

According to the minimal criteria proposed by the International Society for Cellular Therapy in 2006 [24], MSCs must express three positive markers, i.e., *CD105* (*ENG*), *CD73* (*NT5E*), and *CD90* (*THY1*), and lack expression of several negative genes, including *CD45* (*PTPRC*), *CD34*, *CD14* or *CD11b* (*ITGAM*), *CD79a* (*CD79A*) or *CD19*, and *HLA-DR* (*HLA-DRA* and *HLA-DRB1*). When we looked at the expression of those markers in our raw data, we saw the expression of those positive markers (UMI > 0), while negative genes were not expressed (UMI = 0) in most cells (Fig. 1a). Next, we ranked cluster of differentiation (CD) genes by average normalized expression or percentage of cells with at least one UMI across all cells (Additional file 2: Table S2 and Table S3). Classic cell surface markers for MSC definition, including *ENG*, *NT5E*, and *THY1*, as expected, belong to the top 50 highly expressed CDs (Fig. 1b). Among the CDs, integrins, such as *ITGB1*, *ITGA1*, *ITGA2*, and *ITGA5*, which play important roles in MSC morphology, migration, proliferation, differentiation, and survival [47–49], are also highly expressed in WJMSC population (Fig. 1b and Additional file 2: Table S2). To strengthen and validate our scRNA-seq data, we found that most top 100 CDs ranked by average normalized expression in scRNA-seq are also highly expressed in bulk RNA-seq data, including MSC positive markers *ENG*, *THY1*, and *NT5E* (Additional file 2: Table S2). In addition, we assayed the tri-lineage capability of the cultured WJMSCs for scRNA-seq, and the results confirmed that they have the potency to differentiate into osteoblasts, adipocytes, and chondroblasts in vitro (Additional file 3: Figure S1G).

For further analysis, we filtered the outlier cells using the median absolute deviation from the median total library size (logarithmic scale) and total gene numbers (logarithmic scale), as well as mitochondrial percentage for each donor [38]. Totally, 702 outlier cells were removed and 6176 single cells were kept by median absolute deviation method. Considering none or low abundant expressed genes across cells, we also integrated these three data together and removed any gene with an average expression less than 0.1 UMI. Finally, 6176 high-quality single cells with 11,458 expressed genes were passed on to downstream analysis. Across the cells, the number of UMI per cell ranged from 13,121 to 221,432, and the number of genes from 3543 to 9775 (Fig. 1c, d).

### Highly variable genes identified in WJMSCs

**Fig. 2:**
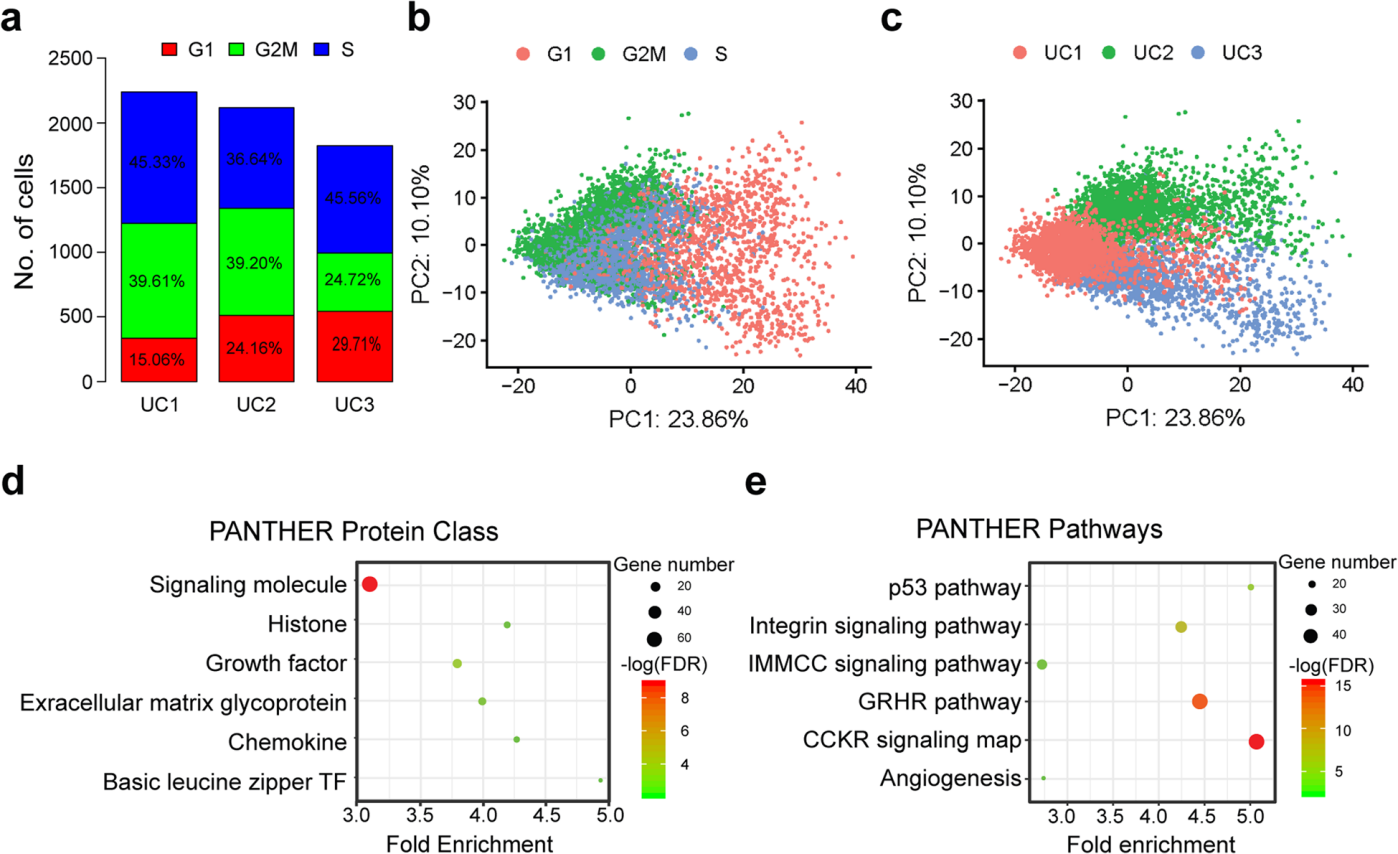
Heterogeneity and highly variable genes in WJMSCs. **a** Phases of cell cycle assigned for each of the three samples. **b**, **c** Cell cycle effects (**b**) and batch effects (**c**) represent the dominant source of heterogeneity in primary cultured WJMSC population. **d** Results of pathway enrichment analysis for highly variable genes identified in WJMSCs. **e** Results of protein class enrichment analysis for highly variable genes identified in WJMSCs. IMMC, inflammation mediated by chemokine and cytokine; GRHR, gonadotropin-releasing hormone receptor

Considering cell cycle effect may influence gene expression, we first assigned cell cycle phases’ state to each cell. The results showed that an average of 22.98%, 34.51%, and 42.51% cells was assigned to G1, G2/M, and S cell cycle phase, respectively (Fig. 2a), suggesting that in vitro cultured WJMSCs are highly proliferated population. Principle components (PCs) analyzed without removing unwanted sources of variation demonstrated that PC1, counting for 23.86% variance, is mainly caused by cell cycle effect (Fig. 2b), while PC2 is counting for 10.10% variance (Fig. 2c), which results from donor-to-donor variation or batch effect. Thus, we selected overlapped highly variable genes among each phase for each donor as mentioned in (Additional file 1) and totally got 770 genes defined as HVGs for the following analysis (Additional file 3: Fig. S2A-D and Additional file 2: Table S4).

Highly variable genes (HVGs) exhibiting high variability across cells represent heterogeneous features within a cell population [50, 51]. Here, we investigated gene functional enrichment of HVGs identified in the WJMSC population. Interestingly, the protein class analysis demonstrated that those genes were overrepresented in signaling molecules, growth factors, extracellular matrix protein, chemokine, histone, and basic leucine zipper transcription factor (Fig. 2d). Besides, the pathway analysis exhibited that these highly variable gene-expressed cross cells were enriched in integrin signaling pathway, inflammation mediated by chemokine and cytokine signaling pathway, gonadotropin-releasing hormone receptor pathway, p53 pathway, and angiogenesis (Fig. 2e). Furthermore, GO enrichment analysis showed that those HVGs are significantly enriched in the extracellular region (Additional file 3: Figure S2E) with binding function, such as protein binding and cytokine receptor binding (Additional file 3: Figure S2G), involved in biological processes like developmental process, signal transduction, cellular component morphogenesis, cell communication, and cell proliferation (Additional file 3: Figure S2F). Micro-environmental interaction is crucial for morphogenesis, cell differentiation, homeostasis, and cell growth [52, 53]. Therefore, variations in the expression of those extracellular functioning genes identified in our analysis could influence interaction of MSCs with micro-environment and cell fate determination [54, 55]. Furthermore, our results showed that highly variable genes in WJMSCs were enriched in distinct biological functions of MSCs, such as anti-inflammation (Fig. 2e), regeneration (Additional file 3: Figure S2F), and wound healing (Additional file 3: Figure S2G), some of which can potentially be selected as candidate markers for a matrix assay to test their therapeutic efficacy in clinical application.

### Characteristics of candidate subpopulations in WJMSCs

To remove batch and cell cycle effects, we scaled the data and performed linear regression to regress the effects out before candidate subpopulation clustering. Here, we used regularized negative binomial regression method to perform normalization and variance stabilization of our scRNA-seq data, which is an appropriate distribution to model UMI count data from a “homogeneous” single-cell population suggested by [56]. The results of nonlinear dimensional reduction performed by UAMP showed that the cells were obviously separated by cell cycle and batch effects before regression, while cells were well mixed after regression and scaling (Additional file 3: Figure S3A, B), implying that those unwanted sources of variation have been effectively removed.

**Fig. 3:**
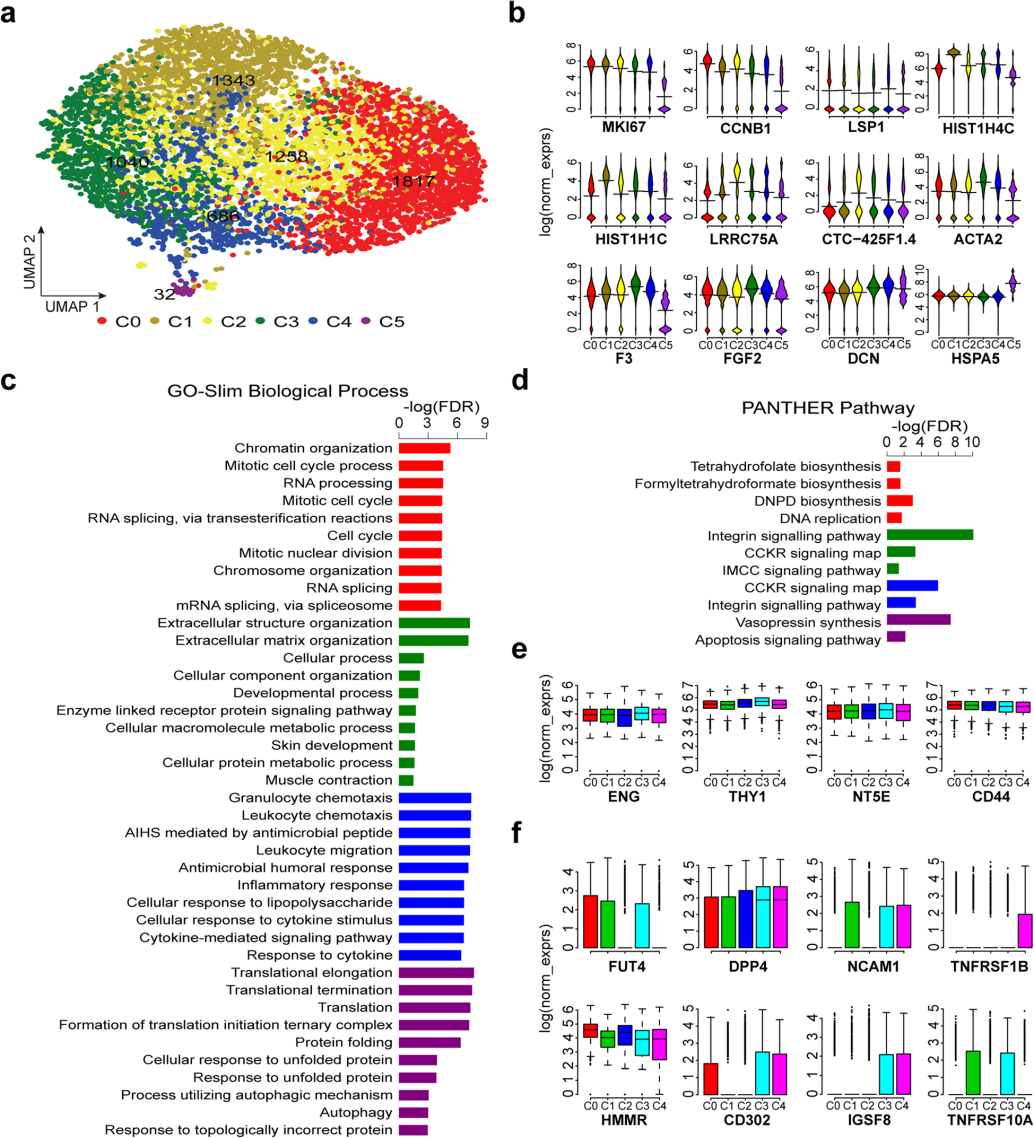
Candidate subpopulations with different functional characteristics. **a** UMAP visualizing the results of cell clustering. **b** Bean plots showing expression of several representative DEGs among the six subpopulations. **c** Pathways significantly enriched for the genes differentially expressed in one subpopulation compared to others. **d** GO-slim biological process enriched for the genes differentially expressed in one subpopulation compared to others. For **c** and **d**, only the top 10 terms with lowest FDR (FDR ≤ 0.05) were present. **e** Boxplots showing expression of classic MSC marker genes in subpopulations. **f** Example of candidate markers showing different expression pattern among the five subpopulations (C0–C4). C0, red; C1, olive; C2, yellow; C3, green; C4, blue; C5, purple. IMCC, inflammation mediated by chemokine and cytokine; DNPD, de novo pyrimidine deoxyribonucleotide

Next, we performed cell cluster analysis by a graph-based clustering approach [57], and six candidate clusters in primary cultured WJMSCs were identified (Fig. 3a). To study the molecular and functional characteristics of these candidate subpopulations in WJMSCs, we performed differentially expressed gene (DEG) analysis among the six clusters (C0–C5) (Additional file 2: Table S5-S6 and Fig. 3b). Intriguingly, *MKI67* (marker of proliferation Ki-67), a gene strongly associated with cell proliferation and growth, is expressed at higher levels in subpopulations C0 and C1 compared with others, implying that subpopulations C0 and C1 possess a higher proliferative capacity. Results of GO enrichment analysis showed that DEGs upregulated in C0 were significantly enriched in DNA replication pathway and cell cycle process as well (Fig. 3c, d). Besides, several histone genes, such as *HIST1H4C* and *HIST1H1C*, exhibited higher expression levels in subpopulations C1 (Fig. 3b). Contrarily, cells in C5 displayed aging characteristics, although a proportion of which is very small in the total populations, and almost all these cells are assigned to G1 phase belonging to UC1 sample (Additional file 3: Figure S3C). We thought that cells in subpopulation C5 may have experienced mutation or replicative senescence during expansion, and they were removed from the following analysis. Across those candidate subpopulations, several markers of MSCs showed a similar expression level (Fig. 3e). Meanwhile, we noted that collagen and chemokine genes across these subpopulations were differentially expressed. Specially, the expression of collagen genes was much higher in C3 while the expression of chemokine genes was higher in C4 (Additional file 3: Figures S3D, E). Furthermore, we presented several candidate surface markers according their differential expression pattern across these candidate subpopulations (Fig. 3f, Additional file 2: Table S6). Whether they could be used efficiently to sort those subpopulations with stable phenotypes and functions needs to be assayed.

**Fig. 4:**
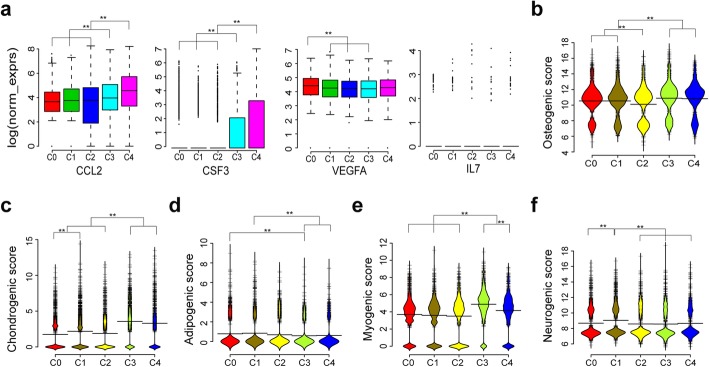
Candidate subpopulations showing different predicted potencies on differentiation and immunosuppression. **a** Boxplots showing expression of genes correlated with PBMC suppression across the five candidate subpopulations (C0–C4). **b**–**f** Bean plots showing distribution of log (norm_exprs) values of osteogenic score (**b**), chondrogenic score (**c**), adipogenic score (**d**), myogenic score (**e**), and neurogenic score (**f**) across the five candidate subpopulations (C0–C4). Wilcoxon rank sum test were performed for significant test, ** *P* < 0.001

In terms of MSC function, on which the MSC clinical application were theoretically based, the DEGs upregulated in subpopulation C3 were enriched in extracellular structure organization, developmental process, skin development, and muscle contraction, while DEGs upregulated in subpopulation C4 were associated with immunomodulation function (Fig. 3c). The secretome analysis revealed that increased levels of some cytokines, such as CCL2, GCSF (CSF3), VEGF, and IL-7, are positively correlated with immunosuppression [58]. Among these subpopulation, the expression levels of *CCL2* and *CSF3* are highest in the C4 subpopulation (Fig. 4a), implicating its immunomodulation therapeutic potential. Besides, the lineage differentiation score among these subpopulations were different, indicating their distinct differentiation propensity to osteogenic, chondrogenic, adipogenic, myogenic, or neurogenic cells (Fig. 4b–f).

To support our data, we isolated CD142^+^ WJMSCs and CD142^−^ WJMSCs by flow cytometric sorting. Considering that CD142^−^ cells account for about 20% of the total cells (Additional file 3: Fig S4A), a comparable number of cells with the highest fluorescence intensity were set as gate for CD142^+^ cells (Additional file 3: Fig S4B). According to our scRNA-seq analysis, *CD142* (*F3*), which is a transmembrane protein and plays multiple important roles in wound healing [59], is expressed at the highest level in subpopulations C3 and lowest in C0 (Fig. 3b). Besides, DEGs, such as *ITGA5*, *MME*, *FGF2*, *SPARC*, *FN1*, *TIMP1*, *COL4A1*, *FLNA*, *COL1A1*, *COL3A1*, and *COL5A1*, which are functionally enriched in skin development and wound healing, were upregulated in C3 compared to others (Fig. 3c, Additional file 2: Table S5). Therefore, the C3 subpopulation is predicted to have greater potency in wound healing. In line with the scRNA-seq data, the RT-qPCR results showed that expression levels of *SPARC*, *COL4A1*, *COL1A1*, and *COL5A1* were higher in CD142^+^ cells (Additional file 3: Fig S4C). Compared to CD142^+^ MSCs, CD142^−^ MSCs showed higher *MKI67* expression, indicating CD142^−^ cells might possess a higher proliferative capacity (Additional file 3: Fig S4C). Indeed, we saw more cells from CD142^−^ MSCs than CD142^+^ MSCs after culturing in vitro for 72 h (Additional file 3: Fig S4D). Then, we compared the wound healing potential between CD142^+^ WJMSCs and CD142^−^ WJMSCs. The results revealed that fibroblasts cultured in conditioned media from CD142^+^ cells presented higher rate of wound closure than the conditioned media from CD142^−^ cells (Additional file 3: Fig S4E and S4F). Therefore, these data demonstrated that CD142^+^ and CD142^−^ WJMSCs showed distinct proliferation capacity as well as “wound healing” potency.

### Single-cell transcriptome comparison between WJMSCs and ADMSCs

**Fig. 5:**
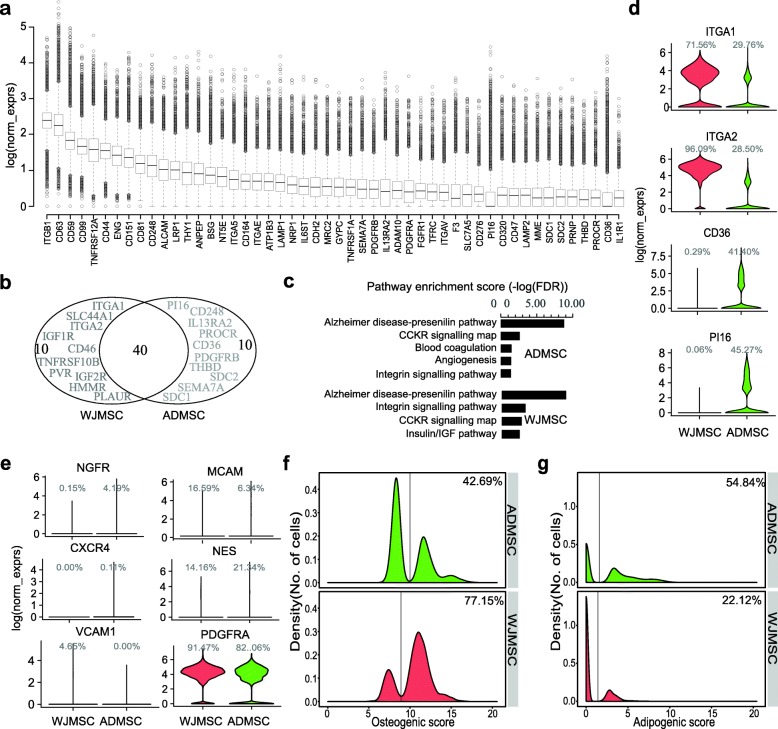
Transcriptome comparison at the single-cell level between WJMSCs and ADMSCs. **a** Boxplot showing the top 50 CD genes ranked by average normalized expression in ADMSCs. **b** Venn diagram showing top 50 ADMSC CD genes overlap with the top 50 WJMSC CD genes, unshared genes were highlighted. **c** Pathway enrichment of top 50 CD genes expressed in ADMSCs and WJMSCs. **d** Example of CD genes showing different expression percentage between ADMSCs and WJMSCs. **e** Violin plots showing expression of markers for reported MSC subpopulations in ADMSCs and WJMSCs. **f**, **g** Density distribution showing osteogenic score (**f**) and adipogenic score (**g**) between ADMSCs and WJMSCs. Percentage indicating proportion of cells assigned to the right side of the line

To provide insights into the heterogeneity of MSCs, several previous studies have compared gene expression of MSCs isolated from different sources using bulk-cell transcriptomic profiles [60–65]. However, even MSCs derived from the same tissue exhibited inter-population functional heterogeneity, such as different differentiation potency and proliferation capacity. Bulk RNA-seq measures the average expression of genes, which is the sum of cell type-specific gene expression weighted by cell type proportions. Bulk transcriptome comparisons may hide some meaningful information that can help to elucidate the underlying mechanisms of functional heterogeneity. Thus, here, we compared the transcriptome data at the single-cell level between WJMSCs and ADMSCs. As expected, a lot of highly expressed classic MSC surface markers are shared between these two MSCs, including *ENG*, *NT5E*, *THY1*, and *CD44* (Fig. 5a, b and Additional file 2: Table S3). Meanwhile, some unshared CDs were identified (Fig. 5b), which suggest a phenotypic diversity between WJMSCs and ADMSCs. These unshared genes are involved in different cell signaling pathways inferred from the pathway enrichment analysis of the top 50 CDs (Fig. 5c). Not surprisingly, some of these unshared CDs, although ranked in the top 50 genes by average expression (Fig. 1a and Fig. 5a), are only expressed at high levels in a small proportion of the MSCs (Fig. 5d). Some of the unshared CDs are expressed (or not expressed) in the majority of the MSCs derived from one tissue but are expressed only in a small proportion of the MSCs in the other one, such as *CD36*, which plays an important role in the formation of intracellular lipid droplets [66], as well as *ITGA1*, *ITGA2*, and *PI16* (Fig. 5d and Additional file 2: Table S3). Those CD genes hold the potential to be used as markers for subpopulation sorting for further physiological and functional research. Accordingly, we also found that several markers, which have been reported to identify special MSC subpopulations with different biological functions, are expressed weakly in a small portion of MSCs, including *CD271* (*NGFR*) [67, 68], *CD146* (*MCAM*) [69], *CXCR4*, *NES* [70], and *CD106* (*VCAM1*) [71], except *PDGFRA*, which are highly expressed in most cells both in WJMSCs and ADMSCs (Fig. 5e and Additional file 2: Table S3).

Increasing reports demonstrated that MSCs derived from different sources exhibited distinct biological properties, such as proliferative capacity, multi-lineage differentiation ability, and immunomodulation potency [72], although they all meet the minimal criteria for defining multipotent MSCs. As regards the differentiation ability, we evaluated it by calculating the lineage differentiation score using single-cell gene expression data from WJMSCs and ADMSCs. Interestingly, density distributions of lineage score displayed two major peaks, while only one peak was observed in housekeeping genes analysis (Fig. 5f, g and Additional file 3: S5A-D), which indicates the existence of multiple subpopulations in MSCs. The density distribution of osteogenic score showed that 77.15% of the cells in WJMSCs have high osteogenic score vs. 42.69% in ADMSCs (Fig. 5f). On the contrary, more cells (54.84%) in ADMSCs have higher adipogenic score than in WJMSCs (22.12%) (Fig. 5g). These results suggested that WJMSCs have the propensity towards the osteogenic lineage while ADMSCs are inclined to differentiate into adipose cells, which are in line with previous studies [33]. Moreover, the difference in other lineage differentiation potencies also existed, such as the chondrogenic and myogenic potential (Additional file 2: Figures S5A, C).

Recently, human skeletal stem cells (SSCs) and adipose progenitor cells were identified [73, 74]. Human SSCs with PDPN ^+^CD146 (MCAM) ^−^CD73 (NT5E) ^+^ CD164 ^+^ phenotype have the ability to generate progenitors of the bone, cartilage, and stroma, but not fat [73]. Human adipose progenitor cells which expressed DPP4 are able to give rise to committed ICAM1^+^ preadipocytes [74]. To determine whether cultured MSCs consist of cells with phenotype as SSCs or adipose progenitors, we analyzed single-cell expression data with the above marker genes. Notably, the proportion of cells which expressed SSC markers in WJMSCs is much higher than in ADMSCs (Additional file 3: Figures S5E, F), while more cells with adipose progenitor’s markers exist in ADMSCs (Additional file 3: Figures S5G, H). These results further indicated that bulk-cell variations among MSCs from different sources may originate from the composition diversity of distinct subpopulations.

## Discussion

MSCs are promising cell therapy products with great potential in promoting tissue regeneration and modulating inflammation. However, significant variations were reported on MSCs that were isolated from different donors and different tissue sites. Unrefined, non-standardized isolation and culture techniques become the challenges of standardization in processes of MSC product manufacturing and quality management. Even in a “homogeneous” population, which is defined by the classic minimal criteria, including cell size [75], morphology [76–78], proliferation capacity [75], differentiation potency [27], and immunomodulation capacity [76, 77], these cells still display phenotype and function heterogeneity among individual cells [79]. In previous clinical trials, functional variation and heterogeneity in MSCs are potentially the main reasons which lead to inconsistent or controversial results [29, 80]. The underlying molecular mechanisms that lead to MSC functional variation and heterogeneity at the cell population level remain unknown, which require further investigation and elucidation.

Recently, several studies have been performed to investigate into the heterogeneity of cultured MSCs by single-cell transcriptomic analysis [35, 81–83]. Huang et al. profiled the transcriptomes of 361 single MSCs derived from two umbilical cords (UC-MSCs) that were harvested at different passages and stimulated with or without inflammatory cytokines. Following the analysis, they concluded that in vitro expanded UC-MSCs are a well-organized population with limited heterogeneity, which is mainly caused by distinct distribution in cell cycle phases [81]. However, the number of cells sequenced for each condition is small (~ 50 cells per condition), and they did not remove the cell cycle effects for the subpopulations identification. Besides, the marker (HMMR) they used to sort the cells to confirm their hypothesis may be unable to isolate different subpopulations. Liu et al. performed a large-scale single-cell transcriptomic sequencing of 24,370 cultured ADMSCs from three donors [35]. They regressed out batch and cell cycle effects before candidate subpopulation classification; however, the results they exhibited in the report were limited to the data analysis pipeline. Wang et al. sequenced a total of 103 single hWJMSCs from three umbilical cords and 63 single hBMMSC cells from two different donors and just focused on gene expression comparison between the two different source-derived MSCs [82]. Thus, the cellular transcriptomic heterogeneity within a MSC population cultured in vitro still has not been comprehensively investigated at the single-cell level.

In this study, we dissected the gene expression heterogeneity of human primary WJMSCs cultured in vitro using scRNA-seq. Single-cell RNA sequencing technologies can offer an unbiased approach for understanding the extent, basis, and function of gene expression variation between seemingly identical cells, revealing complex and rare cell populations, uncovering regulatory relationships between genes, and tracking the trajectories of distinct cell lineages in development [32, 84]. In primary WJMSCs, we found that the HVGs are significantly enriched in extracellular region with binding function, involved in developmental process, signal transduction, cell proliferation, etc. For example, *MKI67*, a marker of proliferation, was identified as one of the HVGs, implying a different proliferate capacity among individual cells. Indeed, we showed that CD142^−^ WJMSCs with higher expression of *MKI67* presented higher proliferation capacity than CD142^+^ WJMSCs cultured in vitro (Additional file 3: Fig S4C). In terms of therapeutic potential, these genes are associated with functional characteristics of MSCs, such as integrin signaling pathway, angiogenesis, and inflammation mediated by chemokine and cytokine signaling pathway (Fig. 2e). Integrin signaling pathway plays a critical role in homing of MSCs to the bone, osteogenic differentiation, and bone formation, and even some integrins are suggested as targets to promote bone formation and repair [85–87]. Several integrin genes were identified in our data with highly variable expression across the cells, such as *ITGA5* and *ITGB1*, which respectively encode α5 and β1 and together form the α5β1 integrin, a cell surface receptor for fibronectin implicated in the control of osteoblastogenesis [87, 88]. Pro-angiogenesis is one of the important biological properties of MSCs, implicating in promoting wound healing and tissue repair [89, 90]. Genes related to the angiogenesis, such as *ANGPT1*, *PDGFRA*, and *VEGFA*., were identified in our HVGs (Additional file 2: Table S4). Studies have reported that *ANGPT1* gene-modified human MSCs could promote angiogenesis and reduce acute pancreatitis in rats [91], while *PDGFRA*^+^ MSCs have enhanced skin repair/regeneration potential [92]. *VEGFA*, and other two cytokines, *CXCL5* and *CXCL8* (IL-8), were required for the angiogenic activity of MSCs and have been selected as an assay matrix for angiogenic potency assay for MultiStem product [93, 94]. Furthermore, in vitro co-culture assays demonstrated that the increased levels of *VEGFA* and chemokine *CCL2* in MSCs were positively correlated with PBMC suppression [58]. Chemokines, a family of small cytokines, are recognized as key mediators of MSCs migration and immunosuppression [8, 95]. Notably, most of the chemokines expressed in primary WJMSCs were highly heterogeneous, including the abovementioned *CCL2*, *CXCL5*, and *CXCL8*. These results indicated that highly variable genes within WJMSCs are associated with classic MSC functional properties and suggested the existence of potential subpopulations with different gene expression patterns.

Although cultured MSCs meet the minimal criteria with classic phenotype, increasing reports demonstrated that many cell surface membrane proteins are not uniformly expressed in MSCs [96]. Several subpopulations with a different phenotype, property, and therapeutic potential have been identified in MSCs derived from different tissues. Some subpopulations express *CXCR4* and have a propensity to migrate to sites of tissue injury [97], while some express *VCAM-1* (*CD106*) and show priority in immunosuppression [71]. However, these above-reported markers only weakly expressed in WJMSCs (Fig. 4e) may be unable to serve as effective markers to isolate these subpopulations in WJMSCs. Here, we classified WJMSCs into several candidate subpopulations (C0–C5) with different functional characteristics. Among these candidate subpopulations, DEG analysis indicated that C0 and C1 show greater proliferation ability while C3 and C4 have greater osteogenic and chondrogenic differentiation potency. Myogenic score is also significantly higher in C3, implying its potential in myocardial repair application. As to immunomodulation, we found that most of the chemokines and some immune-related cytokines detected in WJMSCs upregulate in C4. Furthermore, our analysis demonstrated that DEGs, including *F3* (*CD142*), *ITGA5*, *MME*, *FGF2*, *SPARC*, *FN1*, *TIMP1*, *COL4A1*, *FLNA*, *IGFBP7*, *COL1A1*, *COL3A1*, and *COL5A1*, which are functionally enriched in skin development and wound healing, are expressed at the highest level in the C3 cluster (Fig. 3b). To support our analysis, we isolated CD142^+^ and CD142^−^ WJMSCs to compare their potency in wound healing (Additional file 3: Fig S4). And the results showed that CD142^+^ WJMSCs exhibited lower proliferation capacity and higher wound healing potency than CD142^−^ cells. Many preclinical and clinical studies have been performed to test the regenerative properties of human MSCs in different pathologies, one of which is wound healing [98]. Although the underlying mechanism of MSCs for the enhancement of wound healing remains to be elucidated, several studies have shown that MSCs could secrete some cytokines and growth factors, including *TGFB1*, *FGF2*, *CCL2*, *CXCL8*, and *IGFBP7*, to accelerate cell migration and enhance normal wound healing [46, 99]. Similarly, extracellular matrix (ECM) components, such as collagen types I, V, VI, and XII and fibronectin, along with *SPARC*, play important roles in regulating cell migration as well [46]. Using RT-qPCR assays, we confirmed that most of the abovementioned wound healing-related genes, *TGFB1*, *SPARC*, *COL4A1*, *COL1A1*, and *COL5A1*, were expressed at higher levels in CD142^+^ than in CD142^−^ WJMSCs (Additional file 3: Fig S4C). Taken together, these candidate subpopulations identified in primary WJMSCs would be valuable for further biological characterization via experimental investigations and clinical researches.

ADMSCs can be easily isolated from the stromal vascular fraction (SVF) by enzymatic digestion or non-enzymatic digestion of adipose tissue [100, 101], which are the most widely studied MSCs after BM-MSCs. During wound healing, hair regrowth, and tissue reconstruction, there are several similar bio-molecular pathways that are essential, like angiogenesis, cell migration and recruitment, and cell growth and morphogenesis, which can be enhanced by many growth factors such as TGF-β, FGF family, VEGF, interleukin (IL), PDGF, and IGF. It is well known that these are typical characteristics and biological functions of ADMSCs. Therefore, alone or in combination with hyaluronic acid, platelet-rich plasma (PRP), or fat graft, ADMSCs or AD-SVFs have been tested in many clinical settings to improve wound healing, hair regrowth, and breast reconstruction and demonstrated an encouraging therapeutic potential [98, 102–106]. However, challenges of developing potency assays for those MSC-like products still hinder their clinical applications, which include variability of tissue sources, different isolated and culture methods, largely undefined mechanisms of action in humans, and lack of reference standards [58, 94, 100, 107, 108]. By comparing gene expression between ADMSCs and WJMSCs at the single-cell level, we found that HVGs are largely overlapped between them (Additional file 3: Figure S6A). These shared HVGs include genes, such as *HGF*, *FGF2*, *FGF7*, *VEGFA*, *TGFB2*, *IGF2*, *IGFBP3-7*, *CSF3*, *PDGFA*, *PTGES*, and *PTGS2*, coding proteins which are involved in bio-molecular pathways that could promote cell proliferation, differentiation, and neo-angiogenesis and suppress apoptotic cues, to favor wound healing and hair regrowth processes [98, 105, 109]. Furthermore, functional enrichment analysis of HVGs from ADMSCs showed similar results as those from WJMSCs (Additional file 3: Figures S6B-G), though they exhibited distinct differentiation propensity (Additional file 3: Figure S5). Altogether, we inferred that these HVGs should play critical roles in MSC functional heterogeneity and may serve as candidate markers for further potency association studies. Further studies in cell-to-cell variability in transcriptome, proteome, secretome, and epigenome on MSCs derived from different tissues will increase our understanding of the heterogeneity associated with MSC function and facilitate the development of MSC release criteria for clinical application.

## Conclusions

In summary, our results reveal that highly variable genes within MSCs are significantly enriched in the extracellular region with binding function, involved in developmental process, signal transduction, and cell proliferation. Several candidate subpopulations exhibiting distinct function can be obtained in MSCs applying these HVGs for dimension reduction and clustering. Regarding therapeutic potential, these HVGs are associated with functional characteristics, on which the MSC clinical application was theoretically based, such as development and inflammation response. Altogether, our study suggests that these HVGs hold the potential to be used as candidate markers for further potency association studies.

## Supplementary information

**Additional file 1: Supplemental methods** describing scRNA-seq data analysis for detail, including methods about Quality control, Removal of cell cycle effect, Highly Variable genes identification, Linear and nonlinear dimension reduction, Clustering the cells and Differential expression analysis.

**Additional file 2: Supplemental tables** with six tables. **Table S1.** Marker genes used for potency score analysis. **Table S2**. Top100 CD genes ranked by mean expression in WJMSCs and ADMSCs. **Table S3**. CD genes ranked by percentage of cells expressed the genes (at least one UMI). **Table S4**. List of HVGs identified in WJMSCs. **Table S5.** DEGs in each cluster by comparing it to all of the others. **Table S6.** Results of differential gene expression analysis between two different clusters.

**Additional file 3: Supplemental figures** with five figures. **Figure S1**. Quality of the WJMSCs single-cell RNA-seq data. (A) Number of reads were sequenced for each of the three samples. Percentage of reads mapped to exonic (B) and mapped to transcriptome (C) for each of the three samples. (D) Number of cells obtained for each of the three samples. Boxplot showing number of expressed genes per cell (E) and number of UMI per cell (F) for each of the three samples. (G) Tri-lineage differentiation potency of primary cultured WJMSCs used for scRNA-seq. **Figure S2**. Highly variable genes identification in WJMSCs and GO enrichment analysis. (A) Venn diagram showing overlap of top 2000 highly variable genes among different phases for sample UC1. (B) Venn diagram showing overlap of top 2000 highly variable genes among different phases for sample UC2. (C) Venn diagram showing overlap of top 2000 highly variable genes among different phases for sample UC3. (D) Venn diagram showing overlap of highly variable genes among samples. Results of GO-slim cellular component enrichment analysis (E), GO-slim biological process enrichment analysis (F), and GO-slim functional molecular enrichment analysis for highly variable genes. **Figure S3.** Candidate subpopulations identified in WJMSCs. (A) and (B) UMAP showing dimension reduction before and after batch (A) and cell cycle effect (B) removal. Left, before removal; right, after removal. (C) Histogram showing number of cells for each phase of cell cycle and sample in the candidate subpopulations. (D) Violin plots showing distribution of log normalized expression (log (norm_exprs)) values of collagen genes across the six candidate subpopulations (C0–C5). (E) Violin plots showing distribution of log (norm_exprs) values of chemokines genes across the six candidate subpopulations (C0–C5). **Figure S4**. Wound healing potency for CD142^+^ and CD142^−^ WJMSCs. (A) CD142 analysis by flow cytometry for WJMSCs. (B) Example of gate setting for CD142^−^ (left gate) and CD142^+^ (right gate) cells sorting. (C) qPCR-based expression fold-changes for genes upregulated in C3 plus CCL2, CXCL8 and MKI67 (*n* = 3) between CD142^+^ and CD142^−^ cells. (D) Proliferation for CD142^−^ and CD142^+^ cells cultured in vitro. *n* = 4 for each time point. (E) Representative images of wound healing assays for conditioned media from CD142- and CD142+ cells cultured fibroblast, respectively. (F) Wound closure comparison between CD142^+^ and CD142^−^ cells conditioned media for 24 h (*n* = 9). Data shown are means ± SD, *p < 0.05. Paired two-tailed Student’s t-test were performed for significant test. **Figure S5.** Differentiation potency compared between ADMSCs and WJMSCs. Density distribution showing chondrogenic score(A), neurogenic score (B), myogenic score(C), and housekeeping score (D) between ADMSCs and WJMSCs. Percentage indicating proportion of cells assigned to the right side of the line. (E) Violin plots showing marker genes of SSC expressed in ADMSCs and WJMSCs. (F) Percentage of cells expressed SSC marker genes in ADMSCs and WJMSCs. (G) Violin plots showing marker genes of adipose progenitors expressed in ADMSCs and WJMSCs. (H) Percentage of cells expressed marker genes of adipose progenitors in ADMSCs and WJMSCs. **Figure S6.** Functional enrichment of highly variable genes identified in ADMSCs. (A) Venn diagram showing overlap of HVGs between ADMSCs and WJMSCs. Barplots showing results of GO-slim cellular component (B), GO-slim molecular function (C), Go-slim biological process (D), Reactome pathways (E), Pathways (F), and protein class (G) enrichment analysis for HVGs identified in ADMSCs.

## Data Availability

The data that support the findings of this study have been deposited in the CNSA (https://db.cngb.org/cnsa/) of CNGBdb with accession number CNP0000562. All data analyzed during this study have been included in this published article and its supplementary information files.

## Abbreviations

ADMSCs: Adipose-derived MSCs
CD: Cluster of differentiation
DEGs: Differentially expressed genes
DMEM: Dulbecco’s modified Eagle’s medium
ECM: Extracellular matrix
FBS: Fetal bovine serum
GO: Gene ontology
GvHD: Graft versus host disease
HVGs: Highly variable genes
IBMX: 3-Isobutyl-1-methylxanthine
ISCT: International Society for Cellular Therapy
MSCs: Mesenchymal stem/stromal cells
PCs: Principle components
scRNA-seq: Single-cell RNA sequencing
SSC: Skeletal stem cell
UAMP: Uniform Manifold Approximation and Projection
UMI: Unique molecular identifiers
WJMSCs: Wharton’s jelly-derived MSCs
