# Supplementary material for: Single-cell RNA-seq highlights heterogeneity in human primary Wharton’s jelly mesenchymal stem/stromal cells cultured in vitro
Source: Stem Cell Res Ther. 2020 Apr 6;11:149. doi: 10.1186/s13287-020-01660-4 (PMC7132901; doi:10.1186/s13287-020-01660-4)
Supplement: Supplementary file 1 — Additional file 1: Supplemental methods describing scRNA-seq data analysis for detail, including methods about Quality control, Removal of cell cycle effect, Highly Variable genes identification, Linear and nonlinear dimension reduction, Clustering the cells and Differential expression analysis. [file 13287_2020_1660_MOESM1_ESM.docx]

**Supplemental methods**

**Quality control**

The scRNA-seq data was processed using cellranger-2.0.0 (<https://support.10xgenomics.com>) for each sample with default parameters mapping to the human GRCh38 genome expect the number of recovered cells (--expect-cells option) was set to 2 000. The resulting gene-cell UMI count matrices for each sample were then concatenated into one matrix using the “cellranger aggr” pipeline without normalization.

We filtered outlier cells using the median absolute deviation from the median total library size (logarithmic scale), total gene numbers (logarithmic scale), as well as mitochondrial percentage, as implemented in scran, using a cutoff of 3 (isOutlier, nmads = 3) ([Lun et al., 2016](#_ENREF_3)). In total, 702 cells were removed and 6 176 high quality single cells were passed on to downstream analysis. We also filtered gene expression profile of each cell by mean-based filter. Any gene expressed across all the cells by average UMI less than 0.1 was removed, and 11 458 genes were kept for downstream analysis. Then, clean gene-cell UMI count matrix was loaded as Seurat object ([Macosko et al., 2015](#_ENREF_4)) to manage our dataset for the further analysis. Briefly, UMI counts were used to create Seurat object by CreateSeuratObject function from R package Seurat 3.0 followed by natural log normalization using NormalizeData function with scale.factor parameter set to 1 000 000 as normalized data for genes expression comparison and figures presentation.

SRA files of public available ADMSCs (SRA:SRP148833) scRNA-seq data ([Liu et al., 2019](#_ENREF_2)) were downloaded from <https://www.ncbi.nlm.nih.gov/sra>. Then SRA files were converted to fastq format and analyzed by the same pipeline used for WJMSC single cell transcriptome data.

**Removal of cell cycle effect**

Considering the effect of cell cycle on genes expression, we assigned the cell cycle scores (i.e., G2/M scores and S scores) and phases (i.e. G1, G2/M, and S) for each cell on the basis of scores using function CellCycleScoring from R package Seurat based on the expression levels of a panel of phase-specific marker genes ([Nestorowa and Hamey, 2016](#_ENREF_6)) . To remove the cell cycle effect, the S scores and G2/M scores were used to regress out cell cycle effect.

**Highly variable genes identification**

Taking cell cycle and batch effects on gene expression into consideration, we created sub-datasets of cell each cycle phase for each sample, i.e. 9 sub-datasets from 3 cycle phase of 3 samples, and then performed each sub-dataset respectively for HVGs selection. We used FindVariableFeatures function in Seurat v3 (method="vst") to compute the variance of standardized values across all cells for each gene. This variance represents a measure of single cell dispersion after controlling for mean expression and can be used directly to rank the genes ([Pijuan Sala et al., 2019](#_ENREF_7)). Here, we selected the 2 000 genes with the highest standardized variance as "highly variable" for each sub-dataset, and then chose the overlapped genes among the 9 sub-datasets to define HVGs for the following functional enrichment and dimensional reduction analysis.

**Linear and Nonlinear Dimension Reduction**

Before dimension reduction, We firstly performed normalization and variance stabilization of our data using regularized negative binomial regression ([Hafemeister and Satija, 2019](#_ENREF_1)) with SCTransform in Seurat v3 of argument vars.to.regress to remove confounding sources of variation (variables to regress out including mitochondrial mapping percentage, number of UMI, and cell cycle scores) and argument batch_var to regress out batch effect. Then we used the scaled data of the SCT added as a new assay for the Seurat object to perform dimension reduction**.**

Here, we applied principal component analysis (PCA) and uniform manifold approximation and projection (UMAP) ([McInnes et al., 2018](#_ENREF_5)) for linear and non-linear dimension reduction respectively. PCA was performed by RunPCA function in Seurat library with default parameters, except using HVGs as features augment. We used UMAP to visualize and explore data in two-dimensional coordinates, generated by RunUMAP function in Seurat. The first 20 PCs were used as input.

**Clustering the Cells**

A graph-based clustering approach ([Macosko et al., 2015](#_ENREF_4)) were used to cluster the cells into candidate subpopulations. The first 30 PCs in the data were applied to construct an SNN matrix using the FindNeighbors function in Seurat v3 with k.param set to 20. We then identified clusters using the FindClusters command with the resolution parameter set to 0.6.

**Differential Expression Analysis**

To find differential expressed genes (DEGs) for each subpopulation, Wilcoxon Rank Sum test were performed for significant test using Seurat function FindAllMarkers for every cluster compared to all remaining cells and FindMarkers for distinguishing each other. Genes with average natural log fold change more than 0.2 and FDR less than 0.01 were assigned as DEGs.

**Supplemental Reference**

Hafemeister, C., and Satija, R. (2019). Normalization and variance stabilization of single-cell RNA-seq data using regularized negative binomial regression. bioRxiv, 576827.

Liu, X., Xiang, Q., Xu, F., Huang, J., Yu, N., Zhang, Q., Long, X., and Zhou, Z. (2019). Single-cell RNA-seq of cultured human adipose-derived mesenchymal stem cells. Scientific data 6, 190031.

Lun, A.T., McCarthy, D.J., and Marioni, J.C. (2016). A step-by-step workflow for low-level analysis of single-cell RNA-seq data with Bioconductor. F1000Research 5, 2122.

Macosko, E.Z., Basu, A., Satija, R., Nemesh, J., Shekhar, K., Goldman, M., Tirosh, I., Bialas, A.R., Kamitaki, N., Martersteck, E.M., et al. (2015). Highly Parallel Genome-wide Expression Profiling of Individual Cells Using Nanoliter Droplets. Cell 161, 1202-1214.

McInnes, L., Healy, J., and Melville, J. (2018). Umap: Uniform manifold approximation and projection for dimension reduction. arXiv preprint arXiv:180203426.

Nestorowa, S., and Hamey, F.K. (2016). A single-cell resolution map of mouse hematopoietic stem and progenitor cell differentiation. 128, e20-31.

Pijuan Sala, B., Diamanti, E., Shepherd, M., Laurenti, E., Wilson, N.K., Kent, D.G., Gottgens, B., Stuart, T., Butler, A., Hoffman, P., et al. (2019). Comprehensive Integration of Single-Cell Data. Blood 177, 1888-1902.e1821.
