# Supplementary material for: Single-cell RNA-seq highlights heterogeneity in human primary Wharton’s jelly mesenchymal stem/stromal cells cultured in vitro
Source: Stem Cell Res Ther. 2020 Apr 6;11:149. doi: 10.1186/s13287-020-01660-4 (PMC7132901; doi:10.1186/s13287-020-01660-4)
Supplement: Supplementary file 3 — Additional file 3: Supplemental figures with five figures. Figure S1. Quality of the WJMSCs single-cell RNA-seq data. (A) Number of reads were sequenced for each of the three samples. Percentage of reads mapped to exonic (B) and mapped to transcriptome (C) for each of the three samples. (D) Number of cells obtained for each of the three samples. Boxplot showing number of expressed genes per cell (E) and number of UMI per cell (F) for each of the three samples. (G) Tri-lineage differentiation potency of primary cultured WJMSCs used for scRNA-seq. Figure S2. Highly variable genes identification in WJMSCs and GO enrichment analysis. (A) Venn diagram showing overlap of top 2000 highly variable genes among different phases for sample UC1. (B) Venn diagram showing overlap of top 2000 highly variable genes among different phases for sample UC2. (C) Venn diagram showing overlap of top 2000 highly variable genes among different phases for sample UC3. (D) Venn diagram showing overlap of highly variable genes among samples. Results of GO-slim cellular component enrichment analysis (E), GO-slim biological process enrichment analysis (F), and GO-slim functional molecular enrichment analysis for highly variable genes. Figure S3. Candidate subpopulations identified in WJMSCs. (A) and (B) UMAP showing dimension reduction before and after batch (A) and cell cycle effect (B) removal. Left, before removal; right, after removal. (C) Histogram showing number of cells for each phase of cell cycle and sample in the candidate subpopulations. (D) Violin plots showing distribution of log normalized expression (log (norm_exprs)) values of collagen genes across the six candidate subpopulations (C0–C5). (E) Violin plots showing distribution of log (norm_exprs) values of chemokines genes across the six candidate subpopulations (C0–C5). Figure S4. Wound healing potency for CD142+ and CD142− WJMSCs. (A) CD142 analysis by flow cytometry for WJMSCs. (B) Example of gate setting for CD142− (left gate) [file 13287_2020_1660_MOESM3_ESM.docx]

**Supplemental figures**

**
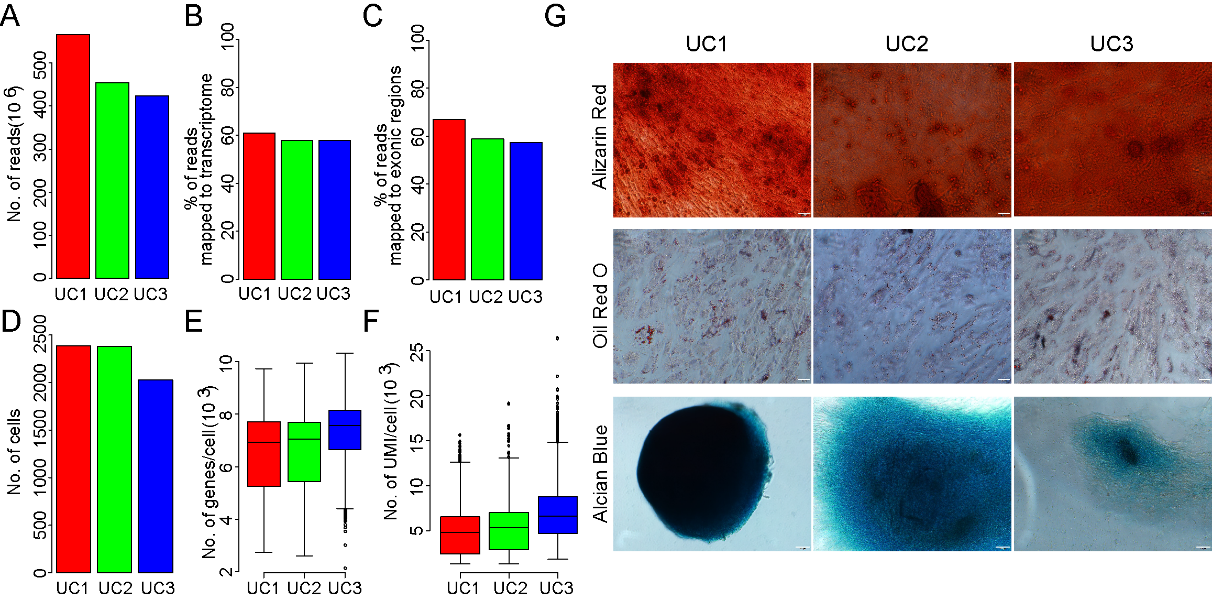
**

**Figure S1. Quality of the WJMSCs single-cell RNA-seq data.**

(A) Number of reads were sequenced for each of the three samples. Percentage of reads mapped to exonic (B) and mapped to transcriptome (C) for each of the three samples. (D) Number of cells were abtained for each of the three samples. Boxplot showing number of expressed genes per cell (E) and number of UMI per cell (F) for each of the three samples. (G) Tri-lineage differentiation potency of primary cultured WJ-MSCs used for scRNa-seq. Alizarin Red, Oil Red O, and Alcian Blue staining indicated the osteogenic, adipogenic, and chondrogenic differentiation, respectively.

**
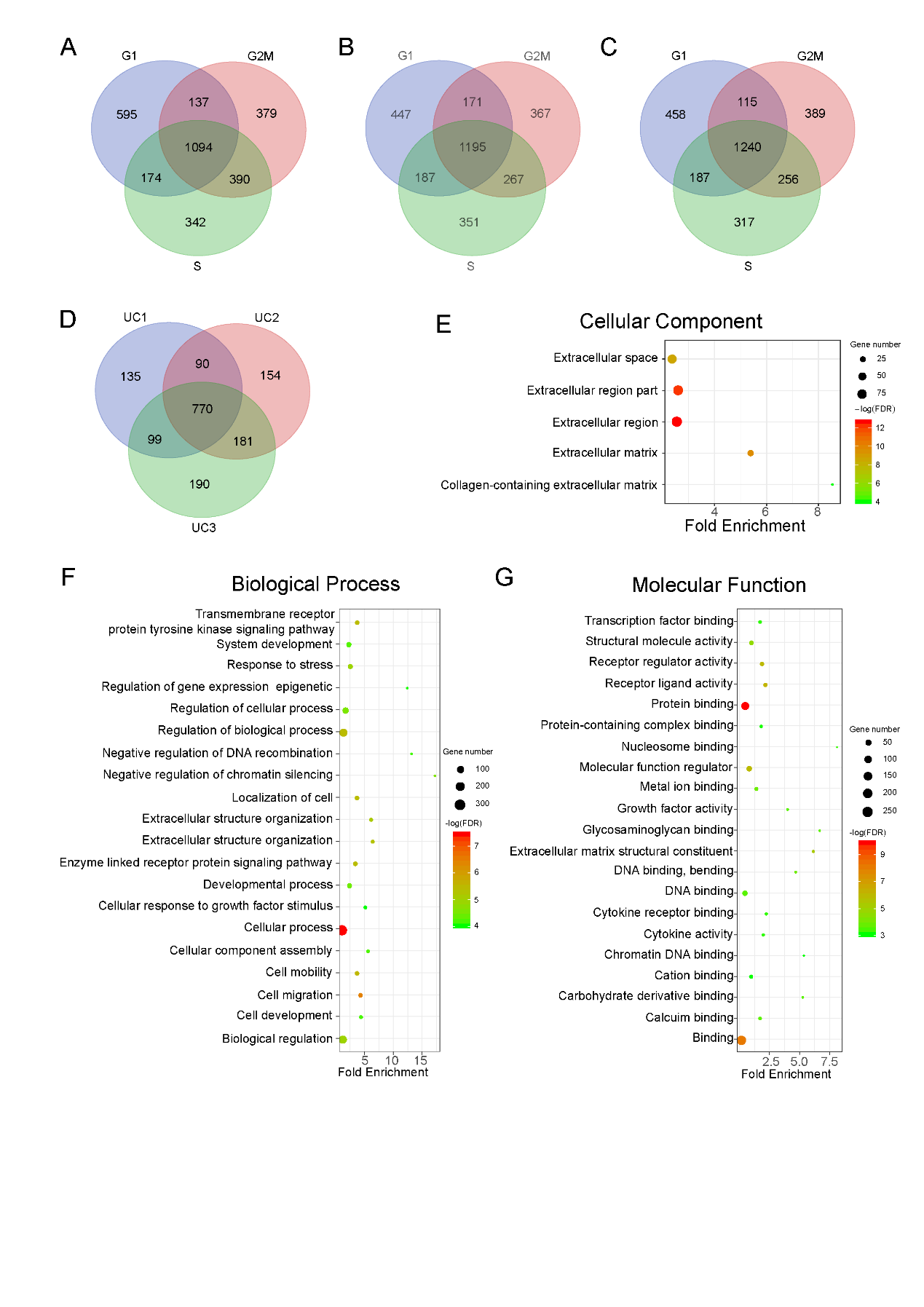
**

**Figure S2. Highly variable genes identification in WJ-MSCs and GO enrichment analysis.**

(A) Venn diagram showing overlap of top 2000 highly variable genes among different phases for sample UC1. (B) Venn diagram showing overlap of top 2000 highly variable genes among different phases for sample UC2. (C) Venn diagram showing overlap of top 2000 highly variable genes among different phases for sample UC3. (D) Venn diagram showing overlap of highly variable genes among samples. Results of GO-slim cellular component enrichment analysis (E), GO-slim biological process enrichment analysis (F), and GO-slim functional molecular enrichment analysis for highly variable genes.

**
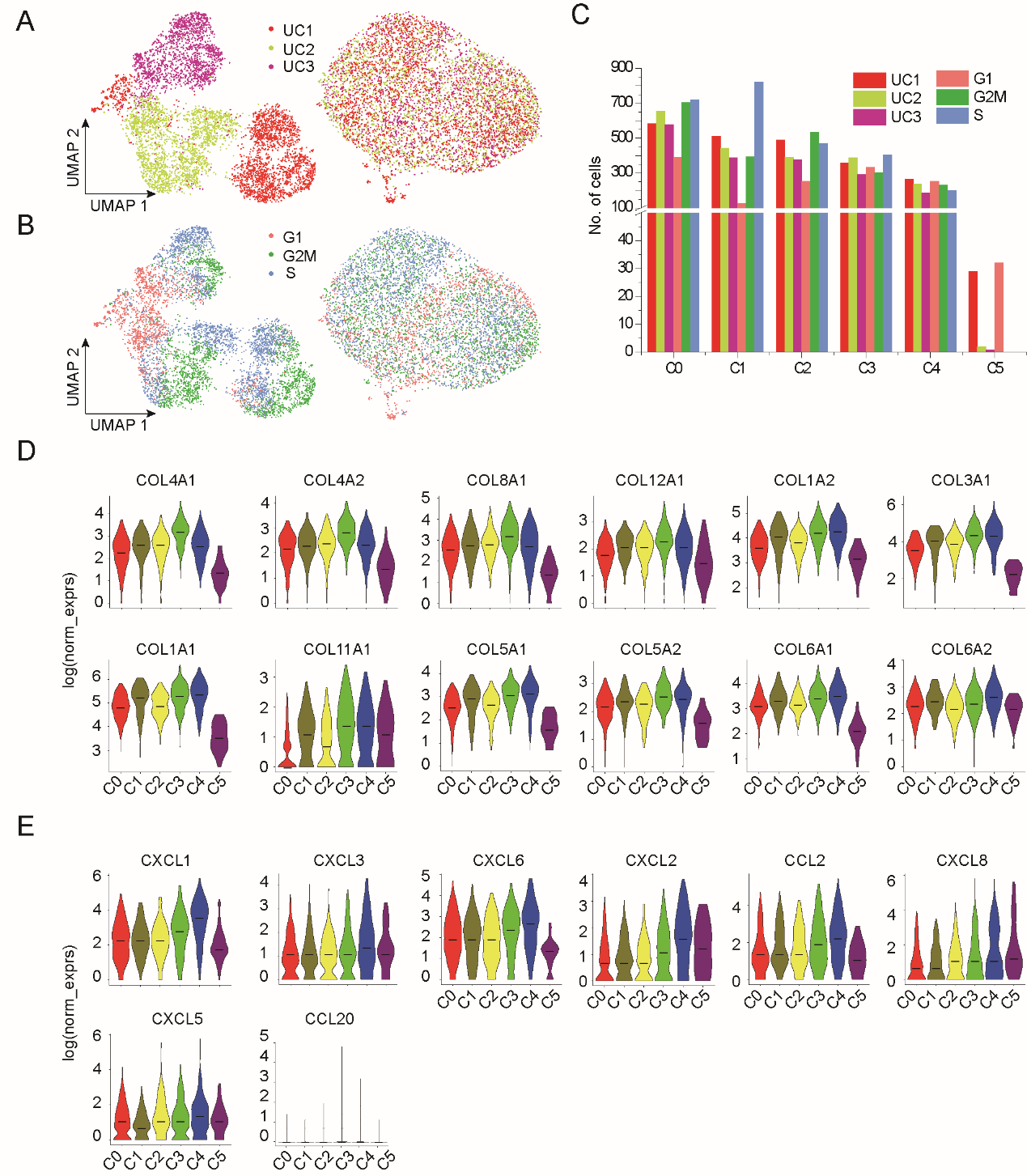
**

**Figure S3. Candidate subpopulations identified in WJMSCs.**

(A) and (B) UMAP showing dimension reduction before and after batch (A) and cell cycle effect removal (B). left, before removal; right, after removal. (C) Histogram showing number of cells for each phase of cell cycle and sample in the candidate subpopulations. (D) Violin plots showing distribution of log normalized expression (log(norm_exprs)) values of collagen genes across the six candidate subpopulations (C0–C5). (E) Violin plots showing distribution of log(norm_exprs) values of chemokines genes across the six candidate subpopulations (C0–C5).


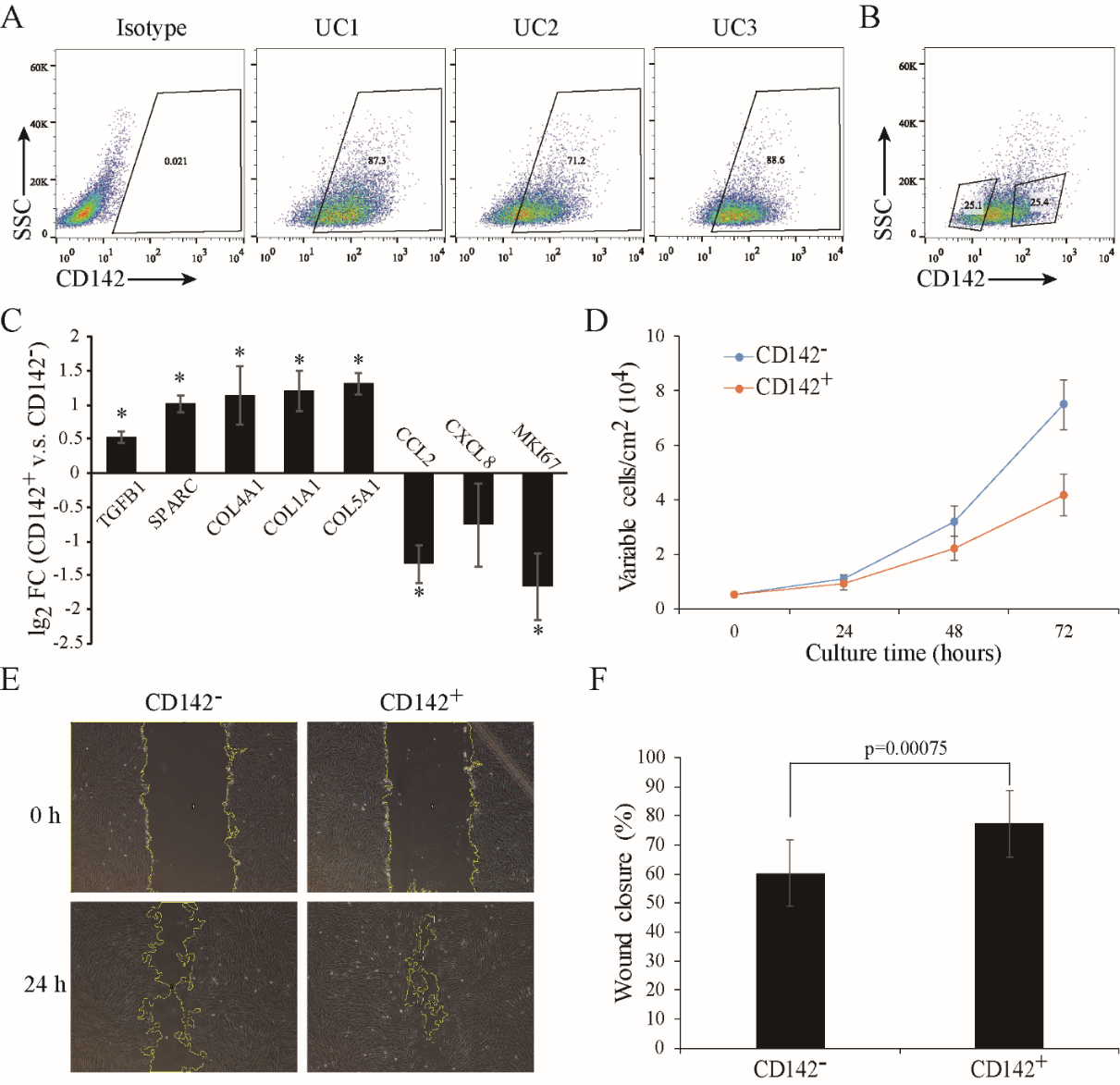


**Figure S4. Wound healing potency for CD142^+^ and CD142^-^ WJMSCs.**

(A) CD142 analysis by flow cytometry for WJMSCs. (B) Example of gate setting for CD142^-^ (left gate) and CD142^+^ (right gate) cells sorting. (C) qPCR-based expression fold-changes for genes upregulated in C3 plus *CCL2*, *CXCL8* and *MKI67* (n=3) between CD142^+^ and CD142^-^ cells. (D) Proliferation for CD142^-^ and CD142^+^ cells cultured in vitro. n = 4 for each time point. (E) Representative images of wound healing assays for conditioned media from CD142^-^ and CD142^+^ cells cultured fibroblast, respectively. (F) Wound closure comparison between CD142^+^ and CD142^-^ cells conditioned media for 24 h (n=9). Data shown are means ± SD, *p<0.05. Paired two-tailed Student’s t-test were performed for significant test.

**
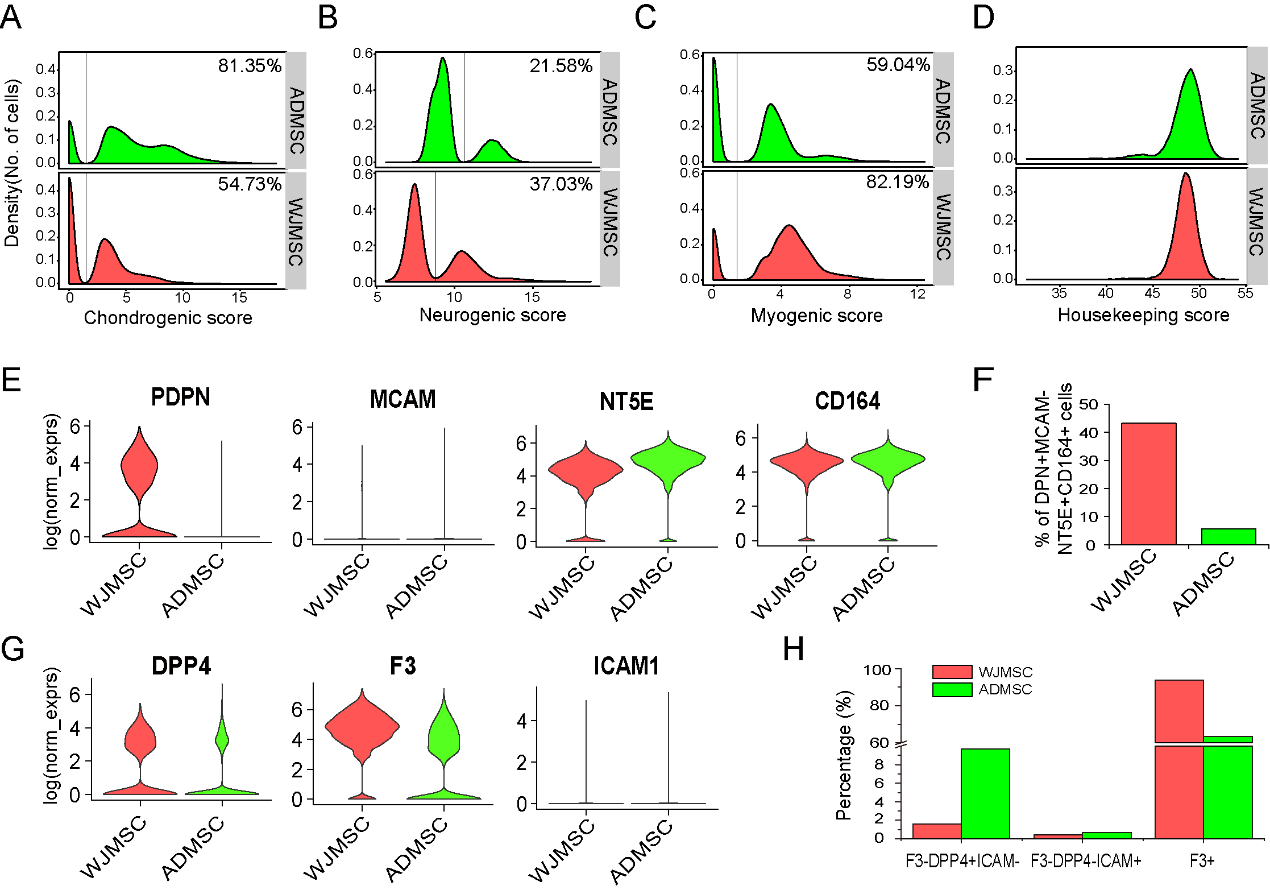
**

**Figure S5. Differentiation potency compared between ADMSCs and WJMSCs.**

Density distribution showing chondrogenic score(A), neurogenic score (B), myogenic score(C), and housekeeping score (D) between ADMSCs and WJMSCs. Percentage indicating proportion of cells assigned to the right side of the line. (E) Violin plot showing marker genes of SSC expressed in ADMSC and WJMSC. (F) Percentage of cells expressed SSC marker genes in ADMSC and WJMSC. (G)Violin plot showing marker genes of adipose progenitors expressed in ADMSC and WJMSC. (H) Percentage of cell expressed marker genes of adipose progenitors in ADMSC and WJMSC. F3-DPP4+ICAM- indicating phenotype of interstitial progenitor cells, F3-DPP4+ICAM- indicating phenotype of committed preadipocytes.

**
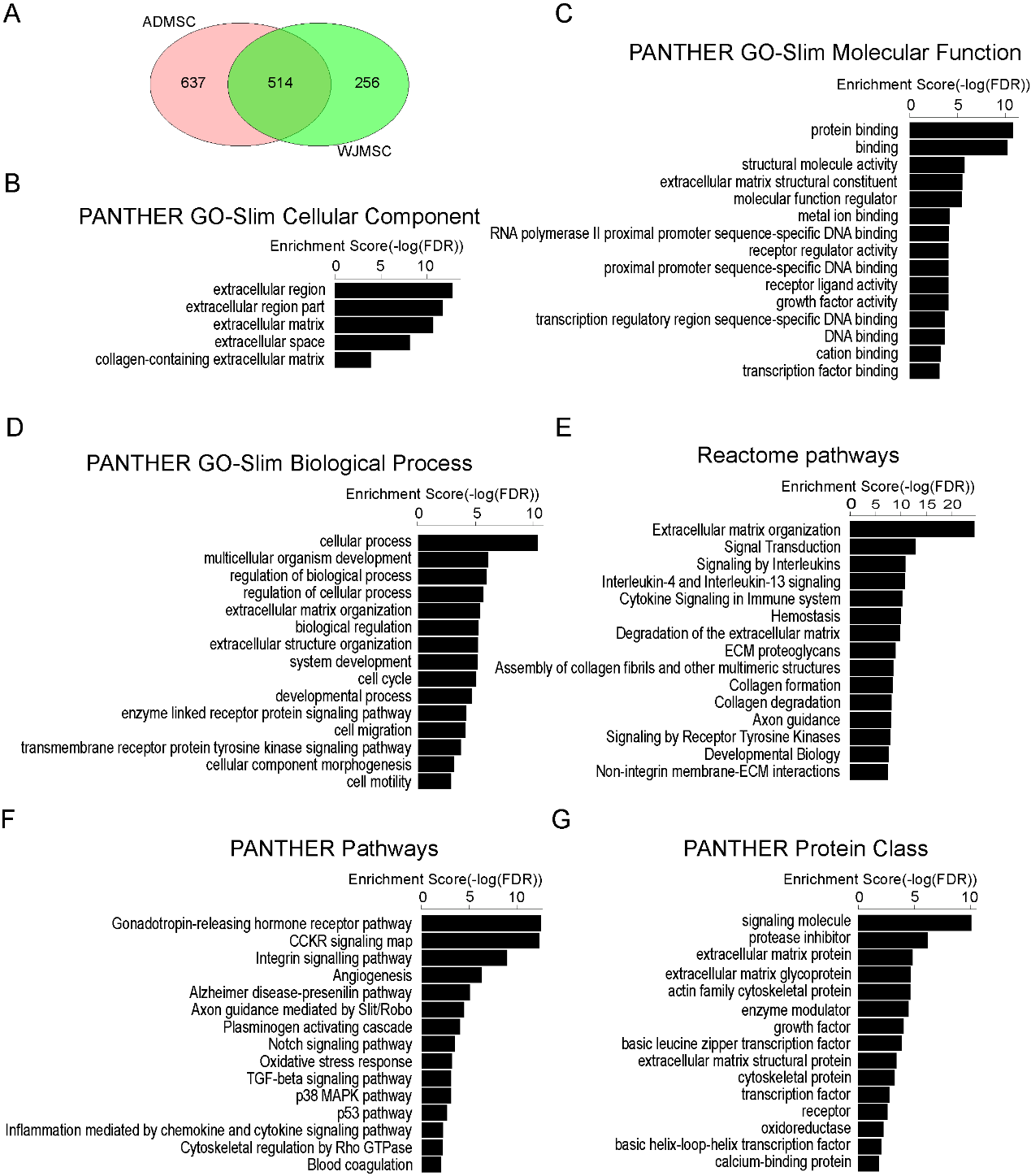
**

**Figure S6. Functional enrichment of highly variable genes identified in ADMSC.**

(A) Venn diagram showing overlap of HVGs between ADMSC and WJMSC. Barplots showing results of GO-slim cellular component (B), GO-slim molecular function (C), Go-slim biological process (D), Reactome pathways (E), Pathways (F), and protein class (G) enrichment analysis for HVGs identified in ADMSC. The top 15 terms ranked by -log (FDR) presented here.
